# Developing a Data Trust Model (Not Only) for Sleep Research: Conceptual Study and Quantitative Survey

**DOI:** 10.2196/66513

**Published:** 2025-12-02

**Authors:** Raphael Jan Dressle, Dieter Riemann, Nicole Thoma, Christina Erler, Rodger Burmeister, Bianka Jogwitz, Katharina Domschke, Kai Spiegelhalder, Joachim Boldt, Svenja Wiertz, Bernd Feige

**Affiliations:** 1 Department of Psychiatry and Psychotherapy, Medical Center - University of Freiburg, Faculty of Medicine, University of Freiburg, Albert-Ludwigs-Universität Freiburg, c/o Medizinische Fakultät, Klinik für Psychiatrie und Psychotherapie, Hauptstraße 5, Freiburg, 79104, Germany, 49 761 270-65570; 2 FZI Research Center for Information Technology, Karlsruhe, Germany; 3 Medical Center - University of Freiburg, Faculty of Medicine, University of Freiburg, Freiburg, Germany; 4 German Center for Mental Health (DZPG), Partner site Berlin/Potsdam, Berlin, Germany; 5 Department of Medical Ethics and the History of Medicine, University of Freiburg, Freiburg, Germany; 6 Center for Philosophy and Ethics of Health, University of Southern Denmark, Odense, Denmark

**Keywords:** health data, data trust, sleep research, informed consent, consent management, digital infrastructure, patient participation, sleep, survey, trust

## Abstract

**Background:**

A large amount of data are generated in health care facilities, yet it is rarely made available for secondary research use. The reasons are manifold. Most importantly, different stakeholders’ needs must be balanced. However, there are currently hardly any feasible solutions for this.

**Objective:**

This study aimed to develop a data trust model with supporting user interface applications to provide a legally and ethically sound framework for secondary use of medical data. The development was based on extensive surveys of various stakeholders.

**Methods:**

Semistructured interviews were conducted with researchers (data users) and institutional representatives of the Medical Center–University of Freiburg, and online questionnaires were administered to patients (data subjects), data users, and institutional representatives. The questionnaire for data subjects covered the dimensions of trust (measured with a 5-point Likert scale), quality of interaction and involvement (measured with a 4-point Likert scale), subjective and objective understanding, and usability (measured with the user version of the Mobile Application Rating Scale). For all other stakeholder groups, the questionnaire focused on usability measured using the user version of the Mobile Application Rating Scale. The surveys comprised a requirement elicitation followed by two rounds of evaluation. Independent-samples Welch *t* tests were used to compare group means between the first and second evaluations.

**Results:**

We devised SouveMed, a framework for secondary use of medical data, applied to the use case of sleep research data. The model includes secure onboarding of data subjects and using digital consent and a digital interface for data users for onboarding, defining research aims, querying the amount of available data, and, finally, either downloading data or having algorithms run on it. At its core is a data trust entity that matches descriptions, consents, and constraints of all stakeholders using digital representations and constraint-solving techniques. Fourteen participants took part in the requirements elicitation, 22 in the first evaluation, and 16 in the second evaluation. In both the first and the second evaluations, data subjects showed a high level of trust in the concept, with mean ratings on the trust scale of 4.23 (SD 0.46) in the first and 4.23 (SD 0.68) in the second evaluation (*t*_15.78_=0.03, *P*=.97). Regarding usability, the mean functionality score of the data user system increased from 3.56 (SD 0.77) in the first to 4.58 (SD 0.38) out of 5 points in the second evaluation (*t*_10.69_=−3.28, *P*=.008). The mean functionality score of the data subject system increased from 4.30 (SD 0.41) in the first to 4.50 (SD 0.74) in the second evaluation (*t*_13.99_=−0.75, *P*=.46).

**Conclusions:**

The SouveMed concept provides a comprehensive framework for the secondary use of medical data. The developed processes can be adapted to other areas of medical research.

## Introduction

Digitalization in health care holds great potential for data-driven research and scientific progress for the benefit of patients and the general public. This includes, for example, automated systems for disease prediction [1-3], as well as innovative approaches that enhance data exchange between practitioners and enable better patient monitoring [4]. However, the use of data for purposes outside the specific treatment context (ie, secondary use) is hindered by several aspects. While an increasing amount of data is being digitally recorded during treatment, the common notion of *data silos*, where data is in principle readily accessible for research purposes, is too simplistic. This is because many processes in routine clinical care are not yet sufficiently digitized, and many analog, paper-based processes remain [5,6]. Furthermore, even digitized data is often not sufficiently preprocessed to be directly accessible for research purposes. They are documented using different software systems that lack interoperability and are stored in a variety of file formats [5-7]. In clinical practice, for example, questionnaires are still frequently handed out in paper form. Digitizing these questionnaires can simply mean that they are scanned and saved in PDF format. A major challenge in this context is the fact that electronic health records, while principally useful for research, are primarily intended to document treatment and reimbursement. Separate measures are necessary to render health record data *fit for purpose* regarding research and to check data quality [8,9].

Legal restrictions are another major obstacle. In the European Union, the introduction of the General Data Protection Regulation (GDPR) has been celebrated as a milestone for the protection of data subjects’ rights but has also been criticized for creating obstacles to research [10-13]. For example, it has been criticized that the GDPR sets higher standards for anonymization than was previously the case in many European Union member states [12,14]. Recital 26 of the GDPR states that pseudonymized data “should be considered to be information on an identifiable natural person.” This has been interpreted to mean that, in principle, all key-coded data must be considered and treated as identifiable personal data, even if the researcher holding the data is not in possession of the key to re-identify it [14]. Therefore, a legal basis is required for the processing of this data, which generally requires greater efforts from researchers and their infrastructure in terms of data protection.

Article 9 of the GDPR outright prohibits the processing of sensitive personal data, which includes health-related data. An exception is made in Article 9 (2) (a) if the patient (data subject) has given explicit consent to the processing of the personal data. Another exception refers directly to scientific research. Under Article 9 (2) (j), processing of sensitive data is allowed for research purposes if appropriate safeguards are in place and a lawful basis for the processing of data is given in the Union or the relevant member state in accordance with Article 6 [11,15].

With regard to consent, there are different consent models to choose from, ranging from broad consent (ie, consent for future studies whose exact research questions and specifications are unknown) to specific consent for a particular study. Hybrid models between broad and specific consent have been developed that allow participants to specify the terms of use without having to give consent for each individual study (tiered consent; for a general overview on different consent models, see [16]). However, in the area of secondary use of medical data, it may be impractical to obtain explicit consent (eg, due to the need to *reconsent* for data already generated), and it has been suggested that it would often be useful to consider other possible legal bases (eg, [11,14]).

The secondary use of health data must, however, also comply with other legal and ethical principles that are not fully taken into account in the GDPR [14]. While from the perspective of the GDPR, obtaining active consent might be replaced by referring to the special purpose of scientific research (if the required legal basis in the relevant member state exists, as mentioned above), consent is still a central ethical standard in medical research. Of particular interest in the field of medical research are the ethical principles formulated in the Declaration of Helsinki (DOH) [17] and Taipei (DOT) [18] of the World Medical Association. The DOH formulates general ethical principles for medical research involving human subjects, while the DOT focuses specifically on principles for databases containing health data. Both statements emphasize the importance of explicit consent for the secondary use of health data. The DOH states that “physicians or other qualified individuals must obtain free and informed consent from research participants for the collection, processing, storage, and foreseeable secondary use of biological material and identifiable or re-identifiable data” [17]. The DOT sets out further rules, such as the right to receive information about the use of the data and the right to change or withdraw consent at any time [18]. Indeed, these principles are in line with the spirit of the GDPR, which formulates and generally strengthens key data subject rights centered on purpose limitation, sovereignty, and transparency, including the right to be informed about processing and the rights to erasure and restriction of processing (see Articles 5 and 6) [10,15,19].

In addition to these basic requirements for the secondary use of research data, it should not be neglected that the secondary use of medical and research data affects many different interests, which must be carefully balanced in order to achieve the acceptance of all stakeholders. Besides the data subject and data users, these stakeholders crucially include the data-generating side, which is typically a clinic or practitioner who cannot waive their care obligations regarding the patient, including the use of sensitive data. With respect to the data subjects, it has been shown that patients are generally very willing to allow access to their health data for public research purposes [20-24]. Nevertheless, many patients are concerned about a lack of privacy, transparency, and data security [25,26]. To address these concerns, a sufficient level of trust is required [27,28]. In this regard, appropriate patient involvement and communication appears to be crucial [25,26]. Adequate information about the secondary use of health data should be provided in an understandable and, at best, personalized form [26], including information about specific projects and findings related to data use [28]. Although there are large interindividual differences in the level of involvement favored by patients, many studies show that a large number of patients prefer a high level of control over their health data [28-32].

As noted above, data users are another important stakeholder group. There is a widespread view that considers health data to be the private property of patients. However, this approach falls short. Concepts of ownership cannot easily be applied to data, and health data can be considered as coconstructed, involving both the data subject and the clinicians and researchers with the generating institution, who contribute their efforts in collecting, classifying, interpreting, processing, and storing the data [33-35]. Because of this crucial contribution and the care obligations of the generating institutions noted above, the needs and constraints of institutions and researchers involved in the generation of health data must be equally considered. Correspondingly, the willingness of institutions and researchers to share health data without appropriate incentives, as well as trust and legal security, is often low [36,37]. At the same time, data users formulate their own requirements for the composition of data sets, utilization options, and acceptable expenditures.

The secondary use of health data, therefore, takes place in a complex environment: legal norms (relating to, but not limited to, data protection regulation), ethical standards, and the interests and needs of different stakeholder groups must be taken into account and balanced. At the same time, a progressive increase in the amount of data available can be expected, which necessitates time- and cost-efficient processes to make use of this data. While initiatives such as the envisaged European Health Data Space (EHDS), the centralized Finnish health data registries, and the German *Medizininformatikinitiative* (medical informatics initiative), among others, prove that the secondary use of health data is on the political and societal agenda, overall, the development is still in its infancy and the great potential that lies in the secondary use of data is currently not being realized. As a possible solution to the problems outlined above, the possibility of data trust models is being discussed (eg, [38-41]). In a report for the United Kingdom government by Hall and Pesenti [41] on the future use of artificial intelligence, data trusts are defined in an overarching manner as “a set of relationships underpinned by a repeatable framework, compliant with parties’ obligations, to share data in a fair, safe and equitable way” (p. 46). Nevertheless, as comprehensively summarized by Lauf et al [42], there is currently no universally accepted definition of a data trust. Instead, based on an analysis of applications across various domains, four *archetypes* of data trusts have been proposed, each with distinct characteristics: data brokerage trustees, data processing trustees, data aggregation trustees, and data custody trustees [42]. In view of the strict data protection requirements associated with the use of health data, existing studies in the medical domain have examined technical features designed to mitigate potential risks. For example, based on a security-by-design approach, the modular separation of demographic and medical data has been proposed to improve data protection within a data trust [43]. In the context of the German medical informatics initiative, the secondary use of medical data is being facilitated by the so-called data integration centers at several university hospitals. Access to the data is controlled by *use and access committees*, which manually review and evaluate data requests before granting access [44,45]. However, beyond these technical and organizational considerations, the successful implementation of a data trust model also depends on stakeholder acceptance and its effective integration into existing clinical workflows.

The aim of the present study was to design a comprehensive legally and ethically sound data trust model from the early conceptualization, through the technical implementation of a prototype system, to its evaluation from the perspectives of various stakeholders. In this study, a data trust model was conceptualized as a neutral entity that facilitates data use while accounting for the interests of all involved stakeholders. Importantly, the data trust—by our definition—does not make decisions on behalf of others. At its core, the system includes a matching service that automatically compares the rules and requirements specified by each stakeholder. When these align, access is granted, without the system itself exercising intentionality. Development prioritized preserving data subjects’ sovereignty while ensuring that system processes remain scalable.

The evaluation was preregistered in a clinical trials registry (German Clinical Trials Registry DRKS00031093). A particular focus was on the use case of sleep research. Sleep research seemed particularly suitable as it is a highly relevant field that warrants further intensified research efforts due to the high prevalence and disease burden of sleep disorders. For example, up to 10% of the general population experiences an insomnia disorder [46] and between 9% and 38% (dependent on age group and methodological approach used) from obstructive sleep apnea [47]. In addition, the field of sleep research is particularly suitable for data-based approaches, as investigating sleep generates a large amount of data, such as sensor data (from the conventionally used polysomnography to modern lifestyle devices such as smartwatches) and subjective data [48]. Besides that, this field is especially sensitive because of its links to psychiatry and is underrepresented in current data-sharing initiatives.

## Methods

### Overview of Study Design

**Figure 1.**
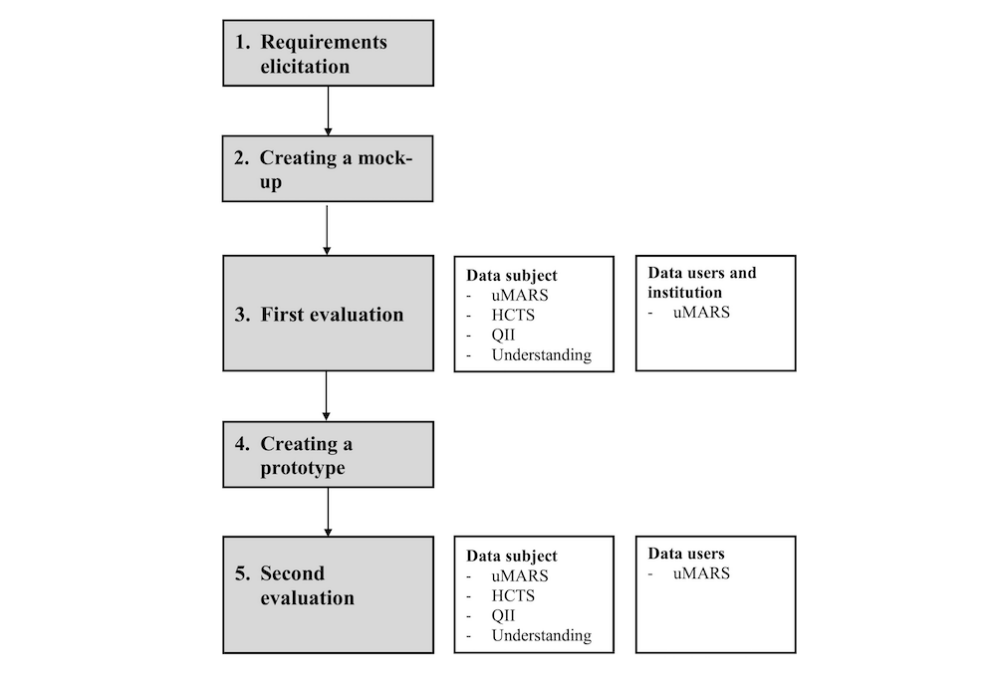
Overview of the study design. HCTS: Human-Computer Trust Scale; QII: Quality of Interaction and Involvement; uMARS: user version of the Mobile Application Rating Scale.

The conceptualization of the data trust model was carried out in five steps (see Figure 1). First, a participatory approach was used to identify relevant needs regarding a data trust model from the perspective of different stakeholders and to define basic requirements for a data trust (requirement elicitation). This included a survey of data subjects, patient representatives, and sleep researchers (data users). An initial conceptual model was then developed, which included a refinement of the relevant procedures and first interactive mock-ups of user interfaces (UI). In a second step, these UIs and procedures were evaluated from the perspective of data subjects, data users, and institutional representatives (including data protection and ethics experts; first evaluation). The feedback was used to further refine the system prototype that was developed in parallel. This prototype was then evaluated again in a second evaluation, which was similar to the process used in the first round of evaluation.

### Requirements Elicitation

Separate questionnaires were developed for data subjects/patient representatives and data users. The questions were developed on the basis of a structured consensus process in the project group with the participation of IT experts and sleep researchers and included questions on general attitudes toward data sharing and preferred consent models as well as technical and organizational requirements, depending on the target group. The full questionnaire used for the requirements elicitation is available upon request.

### Evaluation

#### Overview

The aim of the evaluation study was twofold. First, we wanted to gain some general insights into attitudes toward data sharing with a data trust model from the perspective of different stakeholder groups. These included data subjects from the sleep laboratory, Department of Psychiatry, Medical Center–University of Freiburg, as well as data users/representatives from data-generating institutions. We also aimed at evaluating our specific data sharing process and data trust model from both perspectives. To this end, we conducted a series of anonymous surveys using a semistructured interview and questionnaires (see below). The relevant constructs for the development of the questionnaires were identified in internal project workshops as well as on the basis of a systematic literature search and the needs analysis.

#### Semistructured Interviews

Semistructured interviews were conducted with data users and institutional representatives to gain a more comprehensive picture of the views on data sharing and the corresponding needs. This consisted of a total of 10 predefined questions (see Multimedia Appendix 1).

#### Questionnaire

In total, the questionnaire for data subjects comprised 51 items, which included 4 sociodemographic questions, 5 open questions, 33 Likert scale items, and 9 statement items. The questionnaire for data users/institutional representatives included a total of 30 items, which included 2 sociodemographic questions, 6 open questions, 16 Likert scale items, and 6 statement items.

Trust was assessed using the *human-computer trust scale* (HCTS) [49]. The HCTS is a 12-item questionnaire for assessing user trust based on the *human-computer trust model* [50]. The HCTS includes questions on three dimensions that have been shown to measure trust. These include benevolence (ie, the belief that a system acts in the interest of the user and provides sufficient help if necessary), competence (ie, whether the system has all features and functions that are necessary to fulfill the intended tasks), and perceived risk (individual evaluation regarding the probability of adverse consequences when using the system). All questions were answered on a 5-point Likert scale from 1 (“strongly disagree”) to 5 (“strongly agree”).

Quality of interaction and involvement (QII) was assessed using 7 questions developed by Abdelhamid et al [51]. The questions were translated into German and adapted to relate specifically to the SouveMed process (eg, “Have your feelings and emotions been given the attention they deserve?”). Data subjects had to answer on a 4-point Likert scale (“yes, always” to “no, never”).

The German version of the Mobile Application Rating Scale (MARS) [52] was used to assess usability. Three of four sections that are part of the user version of the MARS (uMARS) [53] were presented. These include the following sections: functionality (eg, “How easy is it to learn how to use the SouveMed app; how clear are the menu labels, icons and instructions?”), aesthetics (eg, “Visual appeal: How good does the SouveMed app look?”), and subjective quality (eg, “Would you recommend the SouveMed app to people who might benefit from it?”). We decided to use only a subset of the sections to avoid overlap with other elements of our comprehensive questionnaire and because some sections were not applicable to the objectives and content of our specific system. All questions were answered on a 5-point Likert scale, with responses reflecting judgments ranging from “inadequate” to “excellent.” Higher scores, therefore, reflect higher quality ratings for the respective domain.

Sociodemographic information and understanding were assessed using 13 items of the questionnaire developed by Richter et al [21]. This included a self-assessment of understanding, subjective reasons for possible poor understanding, and questions to assess the content-related understanding objectively.

### Participants

Current patients and participants of other studies in the sleep laboratory at the Department of Psychiatry, Medical Center–University of Freiburg, as well as data users, that is, sleep researchers and representatives of the Medical Center–University of Freiburg with special expertise in data protection or ethics, were approached and invited to participate voluntarily in the study. Participants who were 18 years or older were included and provided informed consent. We excluded pregnant participants and patients with severe mental or somatic disorders that would have made it unreasonable or impracticable to conduct the survey.

### Ethical Considerations

Approval of the ethics committee of the University of Freiburg, Freiburg, Germany, was obtained (case number 23-1047-S2). The study was conducted in accordance with applicable German and EU data protection regulations as well as the DOH. Participation was voluntary and not compensated. All participants were informed about the aims, methods, risks, and potential benefits of the study and provided documented informed consent. No personally identifying information was collected, ensuring that all study data were fully anonymized.

### Statistical Analysis

The results were averaged, and questionnaire scores are presented as mean (SD) values where possible. Analyses were performed using the statistics software R (version 4.5.0). In the case of missing data, subjects were excluded only from analyses involving the variables for which data were missing (pairwise deletion). Independent-samples Welch *t* tests were used to compare group means between the first and second evaluation, and Hedges *g* was calculated as a measure of effect size. *P* values ≤.05 were considered statistically significant.

## Results

### Requirements Elicitation and the SouveMed Data Trust

#### Requirements Elicitation

We received feedback from a total of 14 participants, including 10 data subjects/patient representatives and 4 data users. From the data subject’s perspective, the survey revealed a clear preference for the possibility of personal contact as part of the informed consent process. There was also a preference for a consent model that allows for further refinement of the conditions under which data sharing is permitted. However, the majority of respondents were critical of giving separate consent for each individual data request. In addition, there was a high general willingness to provide data, although sufficient transparency and data security were often formulated as the most important conditions for this. Furthermore, 5 (50%) of the data subjects considered it “very important” or “important” to receive information about study results that were obtained using their own data. With 6 data subjects (60%), a majority considered it “very important” or “important” to receive a brief description of study projects, and 5 (50%) considered it “very important” or “important” to receive more detailed information about the status of these studies. A large proportion of the data subjects surveyed were therefore interested in comprehensive participation.

From the perspective of data users, there was a clear preference for the European Data Format for the raw polysomnography data. The availability of a range of relevant metadata (eg, number of people, age, diagnosis statistics, etc) was also seen as essential to the attractiveness of the system.

#### The SouveMed Data Trust

**Figure 2.**
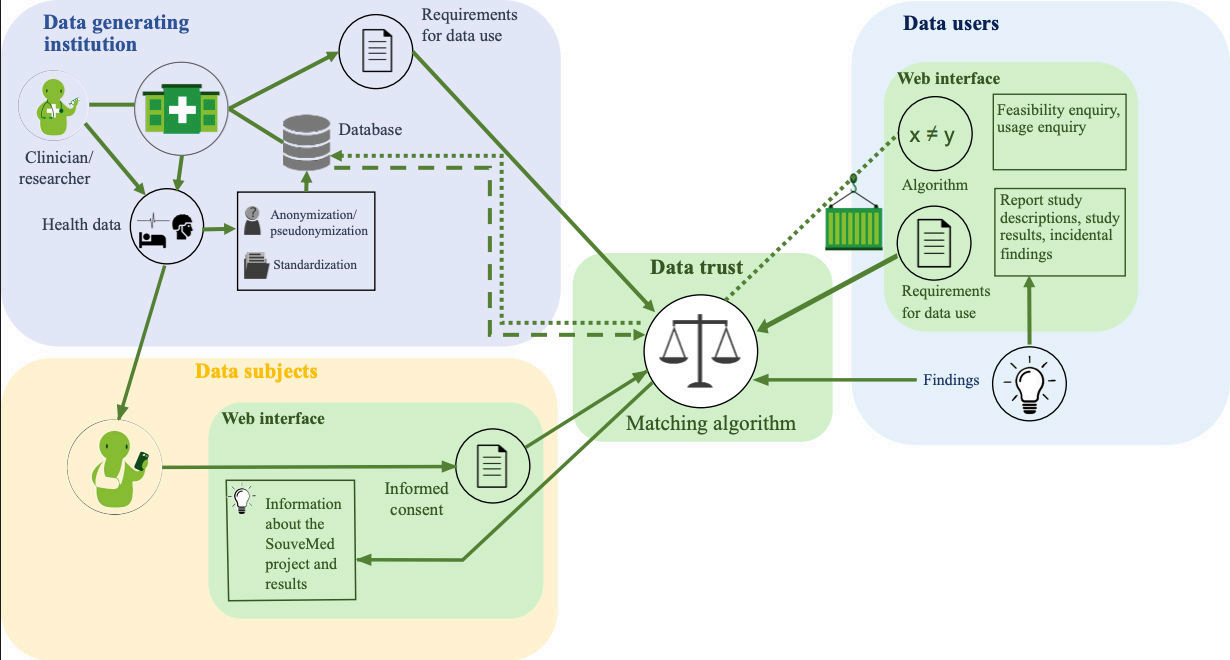
The SouveMed concept.

Based on the needs analysis and workshops by the project group, we developed the *SouveMed* concept, a comprehensive workflow for the secondary use of health data (see Figure 2) with the following key features from the perspective of different stakeholder groups. From the perspective of data subjects, the following functions exist:

- *Onboarding:* A key element of the SouveMed concept is the separation of the onboarding process, that is, the willingness to participate in SouveMed and manage consents with the SouveMed system, from the act of consenting to or disallowing the use of data. It is therefore envisaged that data subjects will only be approached during their visit to the clinical facility to obtain information about SouveMed and to register on the platform. This involves establishing the data subject’s identity and generating a SouveMed ID, that is, a number that can be used together with a password to log in to the platform.
- *Providing consent:* In order to allow participants the necessary time for their decision as well as independence from the clinical setting, decisions are laid down digitally on the SouveMed platform. Consent is designed as a tiered consent (see Multimedia Appendix 2): It applies to a specific visit in the clinical facility and can be further specified, for example, regarding user groups (public or commercial research) and type of shared data (individual or aggregated). The currently valid consent can be viewed at any time on the digital platform, and consent can be withdrawn just as easily as given, amounting to a revocation of data use.
- *Participating in data usage:* The SouveMed platform enables participation in the use of data. Data subjects can obtain information about projects that have accessed their data (including a clear description of the main objectives and results of the studies) and have an overview of current and past consents via a consent archive. Should concerns about data protection or other questions arise, they can easily find the relevant contact information on the SouveMed platform.

From the perspective of data users, the following functions exist:

- *Feasibility inquiry:* Users can submit a feasibility inquiry for a defined research project (ie, specify the inclusion and exclusion criteria and receive the number of available matches with these criteria), as a low-threshold way to determine whether a research project with SouveMed data is realizable.
- *Usage inquiry:* If the result of the feasibility inquiry appears promising, data users can request data usage directly via the SouveMed platform. They can also obtain an overview of their former and current inquiries.
- *Data analysis on the platform*. Users can submit their own scripted algorithms and evaluate datasets in a controlled data trust environment using the curious container technique (for more details, see [54]). This allows for increased data protection, as only aggregated data are shared, including data from participants who only consented to the sharing of aggregated data.
- *Enabling participation of data subjects:* Users can use the SouveMed platform to report incidental findings, as well as provide study outlines and results to data subjects.

Finally, from the perspective of the data-generating institutions:

- Institutions can define requirements for data use, for example, embargos.
- Institutions can monitor the data flow decisions of the platform and ensure conformance with requirements.
- Technical prerequisites were established for this control functionality for data-generating institutions, but it was not implemented as a web interface; SouveMed is designed to be decentralized with a data access component that is under control of the generating institution.

**Figure 3.**
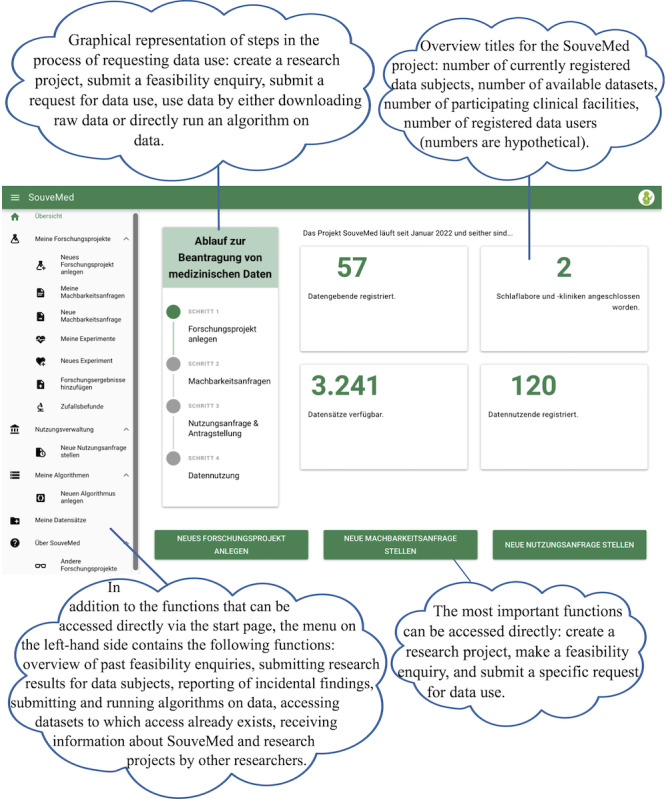
User interface for data users.

**Figure 4.**
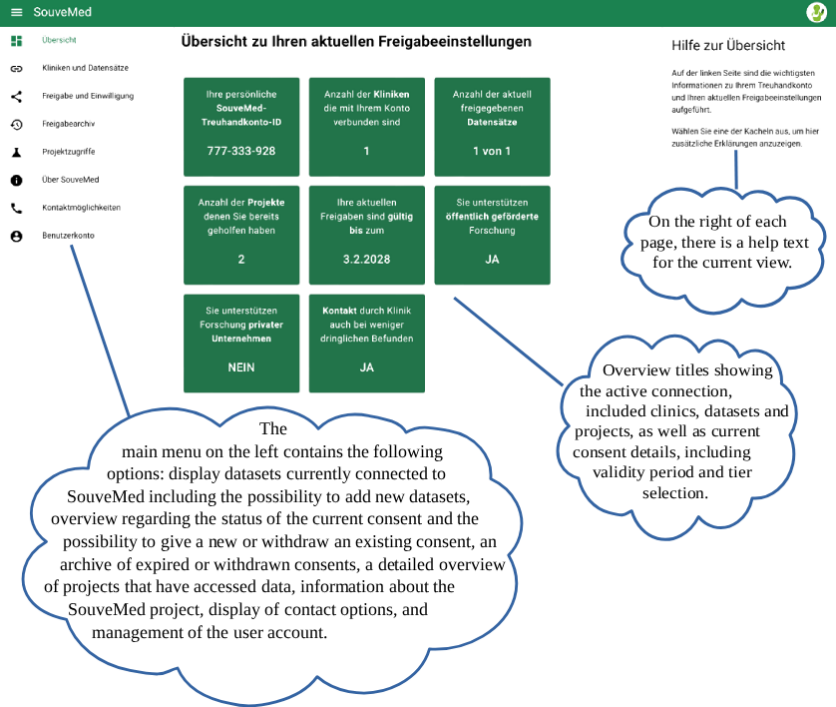
User interface for data subjects.

In the SouveMed project, separate UIs were developed for data users (see Figure 3) and data subjects (see Figure 4). Conceptually, it is intended that data users must create their own user account, whereby a contract between the affiliated institution and SouveMed is a prerequisite for this. A key element of the SouveMed system is the *data trust component*, which automatically matches the descriptions, preferences, and requirements of all stakeholders (data subject, researcher, and institution) using digital contract representations. For details on the technical implementation of the described functions of the SouveMed concept, please refer to [54] and [55].

### Evaluation

#### Sample Characteristics

A total of 22 participants (14 females and 8 males) were studied in the first evaluation. These included 11 patients with sleep disorders and 11 data users/institutional representatives. In the second evaluation, a total of 16 participants (9 females and 7 males) were examined. These included 10 patients with sleep disorders and 6 data users. Of the 22 participants in the first evaluation, 5 (23%) were between 18 and 30 years old, 10 (45%) between 31 and 45 years old, 4 (18%) between 46 and 60 years old, and 3 (14%) between 61 and 75 years old. Of the 16 participants in the second evaluation, 2 (13%) fell into the age range of 18 to 30 years, 7 (44%) into the age range of 31 to 45 years, 5 (31%) into the age range of 46 to 60 years, and 2 (13%) into the age range of 61 to 75 years.

The general willingness to make data available for secondary use was assessed in the data subject group with a mean value of 4.35 (SD 0.58) on a 5-point Likert scale (“very low”=1 point to “very high”=5 points; for the first and second evaluations combined). The need for a corresponding system was assessed in the group of data users/institutional representatives with a mean value of 4.29 (SD 0.77) on a 5-point Likert scale (“very low”=1 point to “very high”=5 points; for the first and second evaluations combined).

#### Semistructured Interview

Data users and institutional representatives described the system as clear and equipped with all the functions they required. It was pointed out that thorough explanations of the secondary use of research data are also important for this stakeholder group. Furthermore, it was uniformly emphasized that the need for a system that facilitates the secondary use of research data is very high.

Previous contact with the concept of data trust models was mentioned by only two participants. With regard to the required functionalities, it was emphasized that access to the system should be easy and that all legal requirements should be met. When the representatives of the institutions were asked whether they would agree to the use of SouveMed in their institution, all gave positive feedback. One person emphasized the importance of an integrated model for all areas of health care in order to avoid isolated solutions for different areas.

#### User Version of the MARS

In the first evaluation, 8 data users/institutional representatives and 10 data subjects provided complete data on the uMARS. In the second evaluation, 6 data users and 10 data subjects provided complete data on the uMARS.

##### Functionality

The mean functionality score of the data user system increased from 3.56 (SD 0.77) in the first evaluation to 4.58 (SD 0.38) in the second evaluation. This difference was statistically significant (*t*_10.69_=−3.28, *P*=.008), with a large effect size (*g*=−1.51, 95% CI −2.76 to −0.26). The mean functionality score of the data subject system increased from 4.30 (SD 0.41) in the first evaluation to 4.50 (SD 0.74) in the second evaluation. This difference was not statistically significant (*t*_13.99_=−0.75, *P*=.46; *g*=−0.32, 95% CI −1.23 to 0.58).

##### Aesthetics

The mean aesthetics score of the data user system increased from 4.08 (SD 0.68) in the first evaluation to 4.17 (SD 0.55) in the second evaluation. This difference was not statistically significant (*t*_11.91_=−0.25, *P*=.80; *g*=−0.12, 95% CI −1.23 to 0.98). The mean aesthetics score of the data subject system increased from 3.93 (SD 0.44) in the first evaluation to 4.23 (SD 0.94) in the second evaluation. This difference was not statistically significant (*t*_12.72_=−0.91, *P*=.38; *g*=−0.39, 95% CI −1.30 to 0.52).

##### Subjective Quality

In the first evaluation, the mean score for the recommendation item (“Would you recommend this app to people who might benefit from it?”) of the data user system was 4.63 (SD 0.52). In the second evaluation, the mean score increased to 4.83 (SD 0.41). This difference was not statistically significant (*t*_11.93_=−0.84, *P*=.42; *g*=−0.42, 95% CI −1.52 to 0.70.

In the first evaluation, the mean score for the recommendation item of the data subject system was 3.90 (SD 0.74). In the second evaluation, the mean score increased to 4.20 (SD 0.79). This difference was not statistically significant (*t*_17.92_=−0.88, *P*=.39; *g*=−0.38, 95% CI −1.28 to 0.53).

In the first evaluation, data users rated the system with an average of 3.75 out of 5 (SD 0.71) stars and in the second evaluation with 4.17 (SD 0.41) stars. This difference was not statistically significant (*t*_11.44_=−1.39, *P*=.44; *g*=−0.65, 95% CI −1.78 to 0.48). Data subjects rated the system with an average of 3.90 (SD 0.57) stars in the first evaluation and 4.10 (SD 0.57) stars in the second evaluation. This difference was not statistically significant (*t*_18.00_=−0.79, *P*=.44; *g*=−0.34, 95% CI −1.24 to 0.57).

### Trust

**Figure 5.**
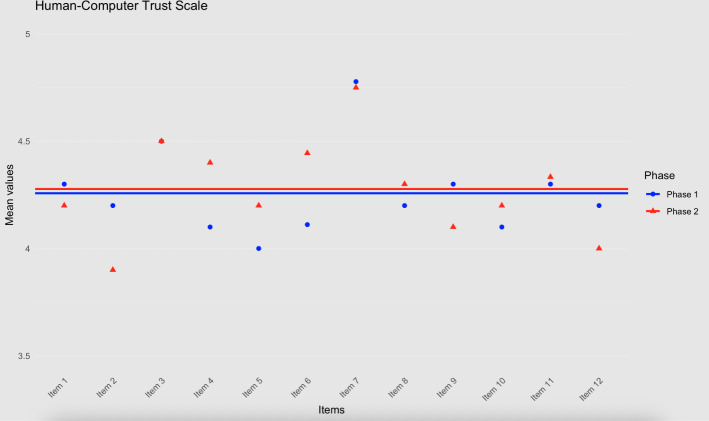
Data subject trust.

In the first evaluation, the assessment of data subject trust resulted in a mean rating of 4.23 (SD 0.46) on the HCTS. One data subject did not respond to the trust scale and was excluded from the analysis. In the second evaluation, the mean rating was 4.23 (SD 0.68; see Figure 5 for item-level mean values). This difference was not statistically significant (*t*_15.78_=0.03, *P*=.97; *g*=0.01, 95% CI −0.87 to 0.91).

### Quality of Interaction and Involvement

**Figure 6.**
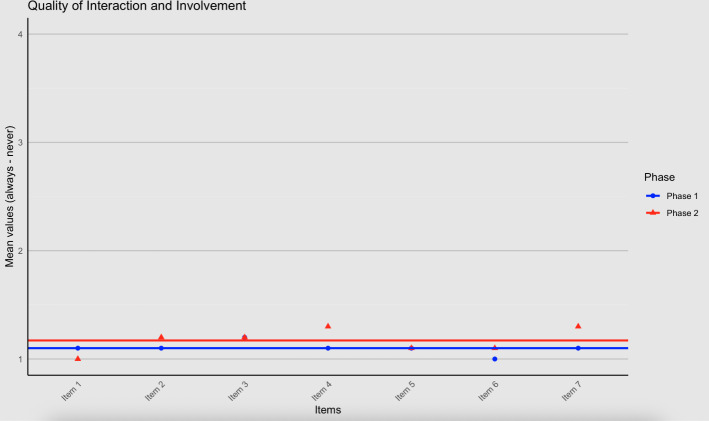
Quality of interaction and involvement.

In the first evaluation, the assessment of QII resulted in a mean rating of 1.10 (SD 0.19), with lower scores indicating higher perceived quality. In the second evaluation, the mean rating was 1.17 (SD 0.34; see Figure 6 for item-level mean values). This difference was not statistically significant (*t*_14.12_=−0.58, *P*=.57; *g*=−0.26, 95% CI −1.15 to 0.66). In the first evaluation, 1 data subject did not respond to the QII scale and was excluded from the analysis.

### Subjective Understanding

When participants were asked whether they thought the information about their data use was understandable, the mean rating was 4.40 (SD 0.97) in the first evaluation and 4.30 (SD 0.95) in the second evaluation. This difference was not statistically significant (*t*_17.99_=0.23, *P*=.82; *g*=0.10, 95% CI −0.80 to 1.00). The scope/length of the information was rated as “just right” by 8 of 11 (73%) data subjects in the first evaluation, “too long” by 3 of 11 (27%) data subjects, and “too short” by none. In the second evaluation, it was rated as “just right” by 8 of 10 (80%), “too long” by 2 of 10 (20%) data subjects, and “too short” by none. The results on objective understanding can be found in the supplementary material (see Multimedia Appendix 3).

### Perceived Risk

Data users and institutional representatives were asked to rate the perceived risk to their institution of using SouveMed. Sixteen (94%) out of 17 responded that they perceived no risk (for the first and second evaluation combined), and 1 of 17 (6%) perceived a risk (for the first and second evaluation combined). Data protection concerns were mentioned as a potential risk.

## Discussion

### Principal Findings

Making health data accessible for secondary research use is a major challenge in the upcoming decades for both health care institutions and research organizations. In addition to the increasing opportunities created by digitalization and the high willingness of data subjects to make health data available, as the present study and other works show [20-23], the potential is not being realized for a number of reasons. These include changing legal norms, inadequate preprocessing of data, and the existence of a large number of different, sometimes conflicting interests of the various stakeholders.

### The SouveMed Concept

Data trust models are a promising solution to address this complexity. However, apart from a few small, heterogeneous initiatives, there are hardly any fully developed approaches in this regard in the field of medical research. We have therefore developed an ethically sound framework for the secondary use of medical data that is applied to the use case of sleep research. The model includes secure onboarding of data subjects and digital use of information, consent, and feedback channels; a digital interface for data users for onboarding, defining research aims and categorization, querying available data set numbers, and, finally, either downloading data or having algorithms run on it; and, crucially, a digital data trust that matches the descriptions and preconditions of all stakeholders (data subject, data user, and institution) using digital contract representations. Data access is granted in our model only when all requirements and conditions set forth by the various stakeholders are satisfied. The explicit focus of our data trust model is on data subject sovereignty and participation, as data subject involvement and appropriate communication are critical to build trust [25,26] and were central to many data subjects in our needs analysis. Accordingly, a majority of data subjects emphasized the importance of a personal contact person in the consent process to be able to ask individual questions. In addition, transparency about data use was mentioned as an important prerequisite for the willingness to provide data, and there was a clear preference for a consent model that allows for further refinement of the conditions under which data sharing is permitted. Based on these results, the SouveMed model provides the opportunity for data subjects to give *real* informed consent by presenting them with comprehensible information material and encouraging them to ask individualized questions in the information process. By separating information and consent in terms of time and space, the ethically questionable linking of health care and the provision of informed consent is avoided. Participants also have the opportunity to receive information about study projects that use their data. The combination of information in face-to-face contact and via a digital platform enables both personalized information for data subjects and patient sovereignty over the amount of involvement, both key requirements demanded of a system for secondary use of health data (eg, [23,26,31]).

SouveMed uses an opt-in solution where data subjects explicitly decide whether or not they wish to share data. This is in line with generally recognized ethical standards for medical research [18,56]. In addition, the present finding regarding the usefulness of tiered consent provided in an electronic format is in line with previous studies investigating different consent models and modes of patient involvement (eg, [30,57-59]). In our approach, the use of a digital interface for consent management also allows data subjects to easily withdraw their consent, which fulfills the requirement in Article 7 of the GDPR that withdrawal of consent must be as easy as giving consent [15].

### Evaluation of the SouveMed Concept

The present evaluation study showed a high degree of trust in our concept in both the first and second evaluations, as measured by the HCTS. It also showed a high level of satisfaction with our communication process, also in the first and second evaluations. Thus, the SouveMed system has proven to be effective in meeting data subjects’ main concerns related to the secondary use of health data. The results of the usability assessment using the uMARS showed very good usability for both evaluations, with a slight increase in usability in the second evaluation. Good usability is key to increasing and maintaining data subjects’ motivation to actively participate in SouveMed and is probably also related to trust [60].

From the perspective of data users and institutions, they may benefit from easy access to raw data and the ability to run algorithms on data in a secure environment. Both our requirements analysis and our evaluation study have shown that there is an urgent need for systems that enable secondary use of research data. The data on uMARS from our evaluation study showed excellent usability in all three subscales (functionality, aesthetics, and subjective quality) from the perspective of data users and institutions, which improved even further in the second evaluation. In this evaluation, all data users surveyed stated that the SouveMed system offers all the functionalities they desire from such a system. Except for a significant increase in the perceived functionality of the data user system, no statistically significant differences were observed between the first and second evaluations. This result is likely due to the limited sample size and the already high ratings in the first evaluation, which constrained the potential for further measurable improvements.

Overall, these results indicate that the SouveMed data trust effectively realizes the needs and requirements identified during the needs analysis and literature review.

### Future Perspectives

Various legislative initiatives in the European Union (eg, EHDS [61]) and in Germany (eg, *Forschungsdatengesetz* [62] and *Gesundheitsdatennutzungsgesetz* [63]) are currently aimed at simplifying the secondary use of health data. This is to be welcomed in principle, as it contributes to the development of a viable research landscape and addresses a major challenge in medical research. Nevertheless, the solutions must be preceded by an analysis of the underlying problems and address them precisely. The results of this and numerous other studies indicate that the secondary use of health data is not hindered by a lack of willingness on the part of data subjects to provide data. It therefore does not seem necessary to pass over data subjects by allowing the use of health data without consent. There is also no lack of infrastructure for centralized data storage, which entails far more risks but only minimal benefits [64]. What is notably absent is a clear workflow for the secondary use of health data that can be easily integrated into routine clinical care, that takes data subjects’ needs into account and thereby gains their long-term acceptance, and that relieves researchers of complex legal considerations and negotiations.

In the European Union, a standardized legal framework has been established through the EHDS, which permits an opt-out mechanism for the secondary use of health data [61]. According to Article 54, this should be implemented via “…an easily understandable and accessible user-friendly mechanism to exercise that right to opt out…” Article 54 further emphasizes that it is “…imperative to provide natural persons with sufficient and complete information regarding their right to opt out…” Nevertheless, how this will be implemented in practice in the individual member states is still largely unclear. Although an opt-in solution is preferred in our project, the SouveMed concept could serve as a blueprint for a system that enables data subjects to receive information about the secondary use of their health data and to express their preferences, including a possible opt-out.

Sleep research was selected as a use case because large volumes of data are typically generated in this field (eg, through polysomnography). In this context, it is important to consider that the likelihood of reidentifying individuals grows with the amount of data collected, making true anonymization increasingly difficult. Similar challenges are expected in other areas of research due to expanding data volumes. Therefore, we argue that the active involvement of data subjects and other stakeholders is becoming ever more critical.

A key advantage of SouveMed is also the algorithmic trust component. Future data exchange will increasingly have to rely on algorithmic decisions to manage large amounts of requests in a time- and cost-efficient manner. Data use and access committees would oversee the rules of data use rather than each single application.

### Limitations

There are some limitations to this study. First, the sample size was small and therefore probably not sufficiently representative. Therefore, surveys of larger patient and other stakeholder groups that include other diagnostic entities are needed. In addition, no *real* implementation of the SouveMed system was carried out as there were insufficient resources to achieve this in the current project. Although we tried to keep the framework of the evaluation as natural as possible, everyone involved was aware that it was not about real data sharing, which could have influenced the results.

Many aspects of our model were developed not only conceptually but also technically (see [54,55]). Nevertheless, further studies focusing on the technical implementation of digital contract representations are needed, as this is a key element of an effectively functioning matching algorithm that was not fully elaborated technically in the SouveMed project.

## Supplementary material

10.2196/66513 Multimedia Appendix 1 Semistructured interview (interview guide, English version).

10.2196/66513 Multimedia Appendix 2 Providing consent via the SouveMed system.

10.2196/66513 Multimedia Appendix 3 Results on objective understanding.

## Abbreviations

DOH: Declaration of Helsinki
DOT: Declaration of Taipei
EHDS: European Health Data Space
GDPR: General Data Protection Regulation
HCTS: Human-Computer Trust Scale
MARS: Mobile Application Rating Scale
QII: Quality of Interaction and Involvement
UI: user interface
uMARS: user version of the Mobile Application Rating Scale

## References

[1] Saberi ZA, Sadr H, Yamaghani MR An intelligent diagnosis system for predicting coronary heart disease.

[2] Nazari M, Emami H, Rabiei R, Hosseini A, Rahmatizadeh S (2024). Detection of cardiovascular diseases using data mining approaches: application of an ensemble-based model. Cogn Comput.

[3] Sadr H, Salari A, Ashoobi MT, Nazari M (2024). Cardiovascular disease diagnosis: a holistic approach using the integration of machine learning and deep learning models. Eur J Med Res.

[4] Nazari M, Moayed Rezaie S, Yaseri F, Sadr H, Nazari E (2024). Design and analysis of a telemonitoring system for high-risk pregnant women in need of special care or attention. BMC Pregnancy Childbirth.

[5] Benson T, Grieve G, Benson T, Grieve G Principles of Health Interoperability: FHIR, HL7 and SNOMED CT.

[6] Pohlmann S, Kunz A, Ose D (2020). Digitalizing health services by implementing a personal electronic health record in Germany: qualitative analysis of fundamental prerequisites from the perspective of selected experts. J Med Internet Res.

[7] Edmondson ME, Reimer AP (2020). Challenges frequently encountered in the secondary use of electronic medical record data for research. Comput Inform Nurs.

[8] Declerck J, Kalra D, Vander Stichele R, Coorevits P (2024). Frameworks, dimensions, definitions of aspects, and assessment methods for the appraisal of quality of health data for secondary use: comprehensive overview of reviews. JMIR Med Inform.

[9] Robertson ARR, Fernando B, Morrison Z, Kalra D, Sheikh A (2014). Structuring and coding in health care records: a qualitative analysis using diabetes as a case study. jhi.

[10] de Hert P, Papakonstantinou V (2016). The new General Data Protection Regulation: still a sound system for the protection of individuals?. Comput Law Security Rev.

[11] Dove ES (2018). The EU General Data Protection Regulation: implications for international scientific research in the digital era. J Law Med Ethics.

[12] Rumbold JMM, Pierscionek B (2017). The effect of the General Data Protection Regulation on medical research. J Med Internet Res.

[13] Vukovic J, Ivankovic D, Habl C, Dimnjakovic J (2022). Enablers and barriers to the secondary use of health data in Europe: General Data Protection Regulation perspective. Arch Public Health.

[14] Peloquin D, DiMaio M, Bierer B, Barnes M (2020). Disruptive and avoidable: GDPR challenges to secondary research uses of data. Eur J Hum Genet.

[15] Regulation (EU) 2016/679 of the European Parliament and of the council of 27 April 2016 on the protection of natural persons with regard to the processing of personal data and on the free movement of such data, and repealing directive 95/46/EC (General Data Protection Regulation). European Union.

[16] Wiertz S, Boldt J (2022). Evaluating models of consent in changing health research environments. Med Health Care Philos.

[17] World Medical Association (2025). World Medical Association Declaration of Helsinki: ethical principles for medical research involving human participants. JAMA.

[18] WMA Declaration of Taipei on ethical considerations regarding health databases and biobanks. The World Medical Association.

[19] Mondschein CF, Monda C, Kubben P, Dumontier M, Dekker A Fundamentals of Clinical Data Science.

[20] Richter G, Borzikowsky C, Lesch W (2021). Secondary research use of personal medical data: attitudes from patient and population surveys in The Netherlands and Germany. Eur J Hum Genet.

[21] Richter G, Krawczak M, Lieb W, Wolff L, Schreiber S, Buyx A (2018). Broad consent for health care-embedded biobanking: understanding and reasons to donate in a large patient sample. Genet Med.

[22] Richter G, Borzikowsky C, Lieb W, Schreiber S, Krawczak M, Buyx A (2019). Patient views on research use of clinical data without consent: legal, but also acceptable?. Eur J Hum Genet.

[23] Garrison NA, Sathe NA, Antommaria AHM (2016). A systematic literature review of individuals’ perspectives on broad consent and data sharing in the United States. Genet Med.

[24] Hutchings E, Loomes M, Butow P, Boyle FM (2021). A systematic literature review of attitudes towards secondary use and sharing of health administrative and clinical trial data: a focus on consent. Syst Rev.

[25] Hutchings E, Loomes M, Butow P, Boyle FM (2020). A systematic literature review of health consumer attitudes towards secondary use and sharing of health administrative and clinical trial data: a focus on privacy, trust, and transparency. Syst Rev.

[26] Cumyn A, Ménard JF, Barton A, Dault R, Lévesque F, Ethier JF (2023). Patients’ and members of the public’s wishes regarding transparency in the context of secondary use of health data: scoping review. J Med Internet Res.

[27] Park YJ, Chung JE (2017). Health privacy as sociotechnical capital. Comput Human Behav.

[28] Damschroder LJ, Pritts JL, Neblo MA, Kalarickal RJ, Creswell JW, Hayward RA (2007). Patients, privacy and trust: patients’ willingness to allow researchers to access their medical records. Soc Sci Med.

[29] Holm S, Kristiansen TB, Ploug T (2021). Control, trust and the sharing of health information: the limits of trust. J Med Ethics.

[30] Broes S, Verbaanderd C, Casteels M, Lacombe D, Huys I (2020). Sharing of clinical trial data and samples: the cancer patient perspective. Front Med.

[31] Cumyn A, Dault R, Barton A, Cloutier AM, Ethier JF (2021). Citizens, research ethics committee members and researchers’ attitude toward information and consent for the secondary use of health data: implications for research within learning health systems. J Empir Res Hum Res Ethics.

[32] King T, Brankovic L, Gillard P (2012). Perspectives of Australian adults about protecting the privacy of their health information in statistical databases. Int J Med Inform.

[33] Liddell K, Simon DA, Lucassen A (2021). Patient data ownership: who owns your health?. J Law Biosci.

[34] Ballantyne A (2020). How should we think about clinical data ownership?. J Med Ethics.

[35] Hummel P, Braun M, Dabrock P (2021). Own data? Ethical reflections on data ownership. Philos Technol.

[36] Perrier L, Blondal E, MacDonald H (2020). The views, perspectives, and experiences of academic researchers with data sharing and reuse: a meta-synthesis. PLoS ONE.

[37] Tenopir C, Allard S, Douglass K (2011). Data sharing by scientists: practices and perceptions. PLoS ONE.

[38] Paprica PA, Sutherland E, Smith A (2020). Essential requirements for establishing and operating data trusts: practical guidance co-developed by representatives from fifteen Canadian organizations and initiatives. Int J Popul Data Sci.

[39] Milne R, Sorbie A, Dixon-Woods M (2022). What can data trusts for health research learn from participatory governance in biobanks?. J Med Ethics.

[40] Hogan WR, Shenkman EA, Robinson T (2022). The OneFlorida Data Trust: a centralized, translational research data infrastructure of statewide scope. J Am Med Inform Assoc.

[41] Hall W, Pesenti J (2017). Growing the artificial intelligence industry in the UK. Australian Policy Online.

[42] Lauf F, Scheider S, Friese J, Kilz S, Radic M, Burmann A Exploring design characteristics of data trustees in healthcare - taxonomy and archetypes. https://aisel.aisnet.org/ecis2023_rp/323/.

[43] Poschen C, Herres B, Knorr K A threat-driven design of a data-trustee infrastructure for medical data.

[44] Prokosch HU, Gebhardt M, Gruendner J (2023). Towards a national portal for medical research data (FDPG): vision, status, and lessons learned. Stud Health Technol Inform.

[45] Semler S, Wissing F, Heyder R (2018). German Medical Informatics Initiative: a national approach to integrating health data from patient care and medical research. Methods Inf Med.

[46] Riemann D, Espie CA, Altena E (2023). The European Insomnia Guideline: an update on the diagnosis and treatment of insomnia 2023. J Sleep Res.

[47] Senaratna CV, Perret JL, Lodge CJ (2017). Prevalence of obstructive sleep apnea in the general population: a systematic review. Sleep Med Rev.

[48] Perez-Pozuelo I, Zhai B, Palotti J (2020). The future of sleep health: a data-driven revolution in sleep science and medicine. NPJ Digit Med.

[49] Gulati S, Sousa S, Lamas D (2019). Design, development and evaluation of a human-computer trust scale. Behav Inf Technol.

[50] Sousa S, Lamas D, Dias P, Zaphiris P, Ioannou A (2014). Learning and Collaboration Technologies Designing and Developing Novel Learning Experiences.

[51] Abdelhamid M, Gaia J, Sanders GL (2017). Putting the focus back on the patient: how privacy concerns affect personal health information sharing intentions. J Med Internet Res.

[52] Messner EM, Terhorst Y, Barke A (2020). The German version of the Mobile App Rating Scale (MARS-G): development and validation study. JMIR Mhealth Uhealth.

[53] Stoyanov SR, Hides L, Kavanagh DJ, Wilson H (2016). Development and validation of the user version of the Mobile Application Rating Scale (uMARS). JMIR Mhealth Uhealth.

[54] Burmeister R, Erler C, Gauger F, Dressle RJ, Feige B (2024). Advancing sleep research through dynamic consent and trustee-based medical data processing. https://www.iaria.org/conferences2024/ICDS24.html.

[55] Erler C, Bauer AM, Gauger F, Stork W (2024). Decision model to design trust-focused and blockchain-based health data management applications. Blockchains.

[56] (2013). WMA Declaration of Helsinki – ethical principles for medical research involving human subjects. The World Medical Association.

[57] Spencer K, Sanders C, Whitley EA, Lund D, Kaye J, Dixon WG (2016). Patient perspectives on sharing anonymized personal health data using a digital system for dynamic consent and research feedback: a qualitative study. J Med Internet Res.

[58] Wiertz S, Boldt J (2024). Ethical, legal, and practical concerns surrounding the implemention of new forms of consent for health data research: qualitative interview study. J Med Internet Res.

[59] Budin-Ljøsne I, Teare HJA, Kaye J (2017). Dynamic consent: a potential solution to some of the challenges of modern biomedical research. BMC Med Ethics.

[60] Casaló LV, Flavián C, Guinalíu M (2010). Generating trust and satisfaction in e-services: the impact of usability on consumer behavior. J Relationship Marketing.

[61] (2025). Regulation (EU) 2025/327 of the European Parliament and of the council of 11 February 2025 on the European Health Data Space and amending directive 2011/24/EU and regulation (EU) 2024/2847. European Union.

[62] (2024). Eckpunkte BMBF Forschungsdatengesetz. Bundesministerium für Bildung und Forschung.

[63] (2024). Gesetz zur verbesserten Nutzung von Gesundheitsdaten. Bundesrepublik Deutschland.

[64] Hallock H, Marshall SE, ’t Hoen PAC (2021). Federated networks for distributed analysis of health data. Front Public Health.

