# Supplementary material for: Developing a Data Trust Model (Not Only) for Sleep Research: Conceptual Study and Quantitative Survey
Source: JMIR Hum Factors. 2025 Dec 2;12:e66513. doi: 10.2196/66513 (PMC12671904; doi:10.2196/66513)
Supplement: Multimedia Appendix 1 [file humanfactors-v12-e66513-s001.docx]

**Semi-structured interview**

1. Please describe your first impression of the system.
2. What general need do you see to create opportunities for data exchange between institutions?
3. Have you had any previous contact with data trust models?
4. What functionalities do you think an ideal data trust model should have?
5. Would you authorise the use of the system in your institution? (*Question to be asked only to relevant decision-makers)*
6. Does the system have all the functionalities you would like to have? If no: what is missing?
7. How secure do you think the system is in terms of data protection?
8. How transparent do you think the security guidelines are?
9. Were there any functions that were not explained to you? If yes: which ones?
10. Would you like to have direct contact with research projects or is contact with research institutions via the sleep centres sufficient?
