# Supplementary figures and images for: Developing a Data Trust Model (Not Only) for Sleep Research: Conceptual Study and Quantitative Survey
Source: JMIR Hum Factors. 2025 Dec 2;12:e66513. doi: 10.2196/66513 (PMC12671904; doi:10.2196/66513)

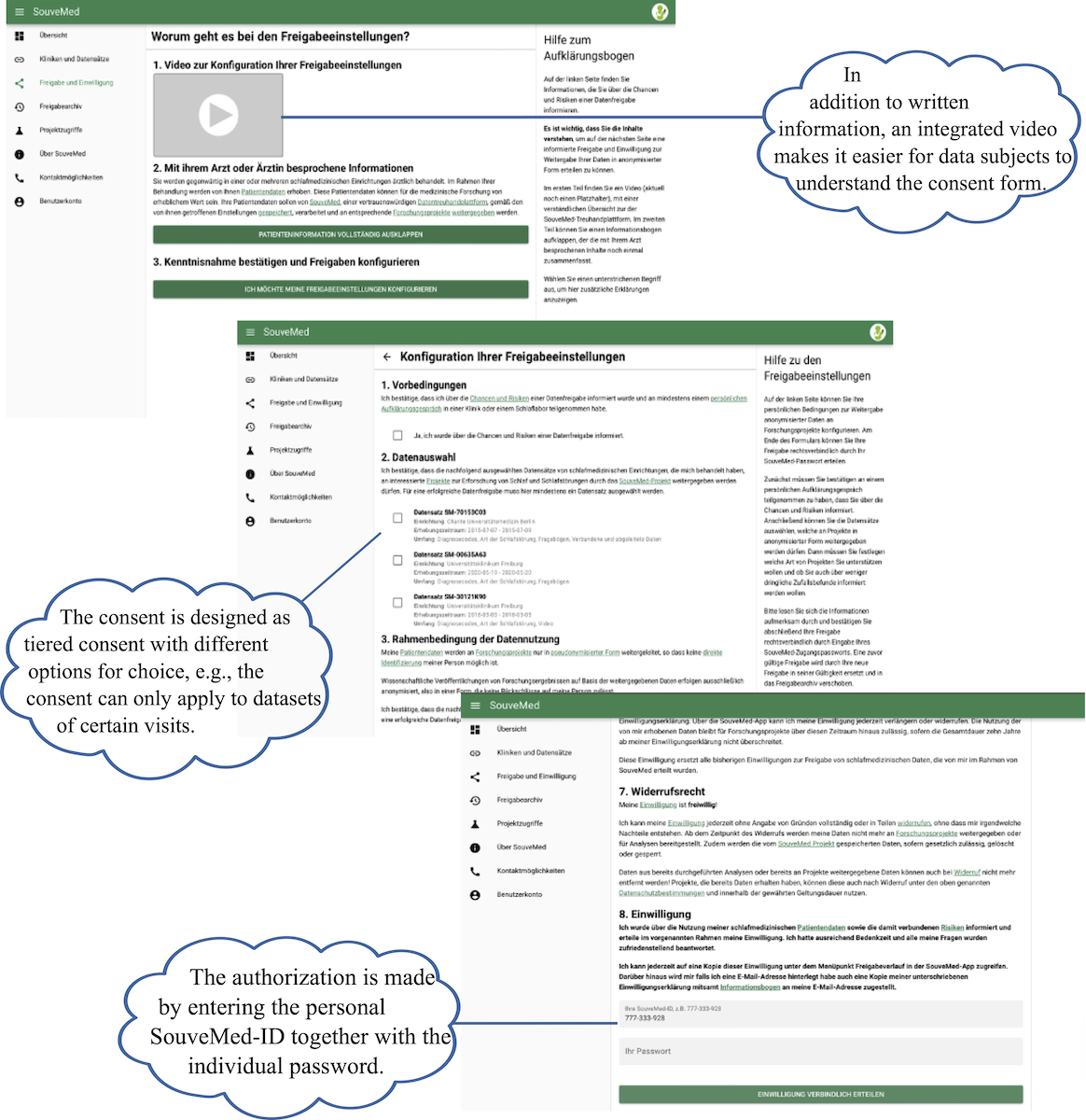

Supplement: Multimedia Appendix 2 [file humanfactors-v12-e66513-s002.png]

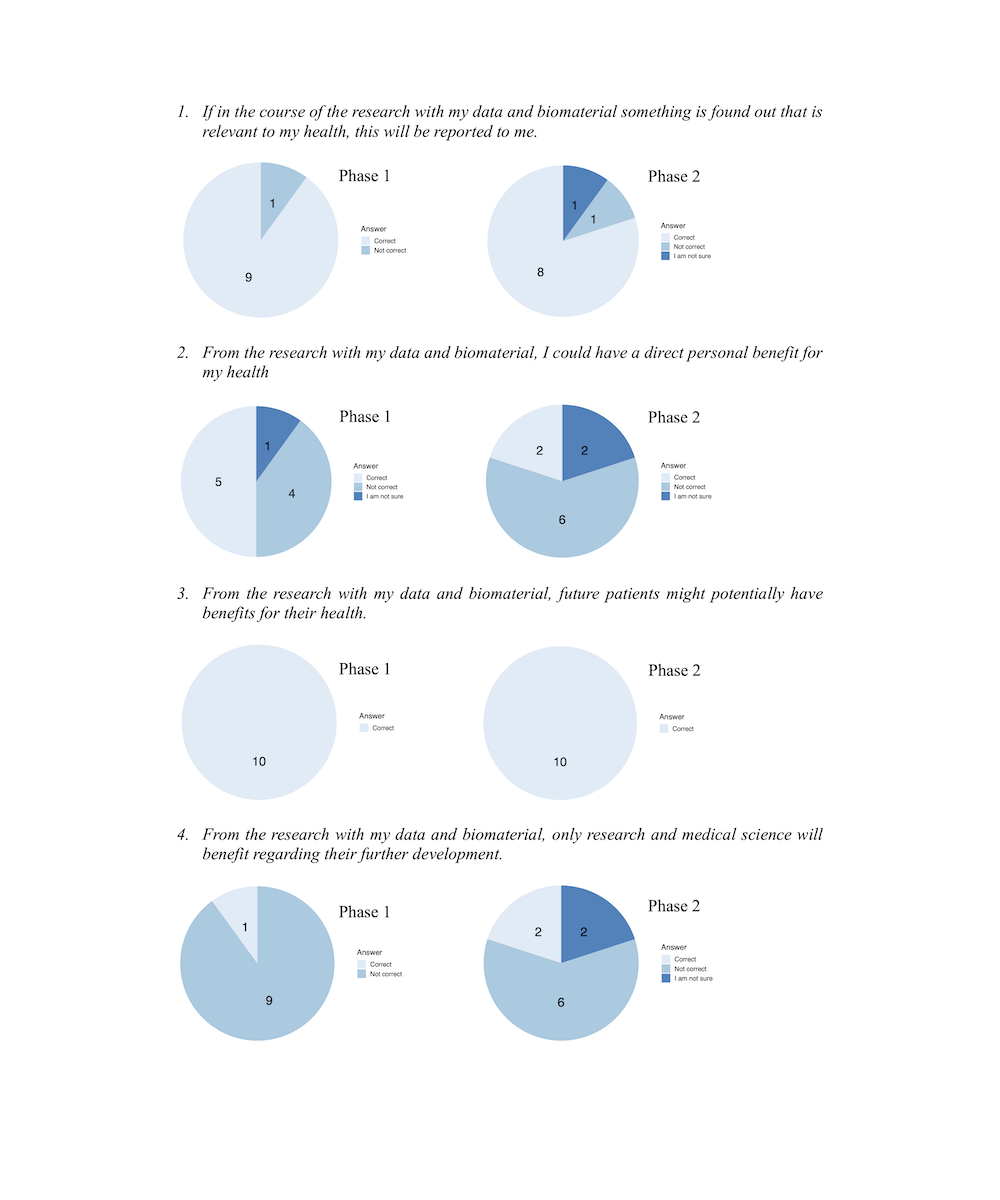

Supplement: Multimedia Appendix 3 [file humanfactors-v12-e66513-s003.png]
